# Cost-effectiveness analysis of Toripalimab regimen for extensive-stage small-cell lung cancer in China and America

**DOI:** 10.3389/fimmu.2025.1556100

**Published:** 2025-05-16

**Authors:** Jing Yang, Fang Chen, Junlin Li, Yujie Zhou, Hao Wang, Yunchun Long

**Affiliations:** ^1^ Department of Pharmacy, Chengdu Second People’s Hospital, Chengdu, Sichuan, China; ^2^ Department of Pharmacy, The First Affiliated Hospital of Xiamen University, Xiamen, Fujian, China; ^3^ Department of Pharmacy, Nanan People's Hospital of Chongqing, Chongqing, China; ^4^ Department of Respiratory and Critical Care Medicine, Nanjing Drum Tower Hospital, Nanjing, Jiangsu, China; ^5^ Department of Pharmacy, Nanjing Drum Tower Hospital, Nanjing, Jiangsu, China; ^6^ School of Basic Medicine and Clinical Pharmacy, China Pharmaceutical University, Nanjing, Jiangsu, China

**Keywords:** cost-effectiveness analysis, extensive-stage small-cell lung cancer, Toripalimab, chemotherapy, EXTENTORCH study

## Abstract

**Objectives:**

Toripalimab combined with chemotherapy is a clinically valuable and important regimen in the treatment of extensive−stage small−cell lung cancer (ES-SCLC). However, there are no studies on the cost-effectiveness of this regimen, so this study was designed to evaluate the cost-effectiveness of Toripalimab regimen for the treatment of ES-SCLC from the perspectives of the Chinese health system and the U.S. health system, respectively.

**Methods:**

A partitioned survival model was developed to simulate the clinical progression and cost consumption of ES-SCLC using the results of the EXTENTORCH study as a source of survival data and incorporating direct medical costs. Model output metrics included incremental cost-effectiveness ratio (ICER), quality-adjusted life-years (QALYs), incremental QALYs, total costs, and incremental costs. The cost-effectiveness of the Toripalimab scheme was judged by comparing the ICER with the willingness to pay (WTP). The robustness of the model was verified by sensitivity analysis and scenario analysis.

**Results:**

The results of the basic analysis showed that from the perspective of the Chinese health system, the Toripalimab group gained 0.18 QALYs more at a cost of $5,204, with an ICER of $29,139/QALY (<WTP). From the standpoint of the U.S. health system, the Toripalimab group spent $156,923 more and also gained 0.17 QALYs more, but the ICER ($915,965/QALY) was much higher than the WTP. Sensitivity and scenario analyses showed the model to be generally stable.

**Conclusions:**

Compared with chemotherapy, the Toripalimab regimen for the treatment of ES-SCLC is cost-effective from the perspective of the Chinese health system, but not from the perspective of the US health system.

## 1 Introduction

Global cancer statistics show that in 2022, there were 2.48 million new cases of lung cancer, accounting for 12.4% of the total number of new cancer cases, and 1.8 million new lung cancer deaths, accounting for 18.7% of the total cancer deaths, which makes lung cancer the malignant tumor with the highest morbidity and mortality rate in the world (1). In China, lung cancer is also the top malignant tumor in terms of incidence and mortality (1, 2). In the US, lung cancer is the third most common cancer and the first in terms of mortality (1). Small-cell lung cancer (SCLC) is a highly aggressive subtype of lung cancer, accounting for 15%-17% of the total incidence of lung cancer (3, 4). SCLC exhibits rapid growth, a high degree of malignancy, and a propensity for metastasis. Nearly 70% of patients are in extensive stage when diagnosed, and are identified as having extensive-stage small-cell lung cancer (ES-SCLC), with a 5-year survival rate of less than 7% (5–7).

Before 2019, platinum-based DNA cross-linking agents (such as cisplatin or carboplatin) in combination with topoisomerase inhibitors (such as etoposide or irinotecan) is the preferred chemotherapy regimens for ES-SCLC (5). Although the short-term efficacy of this combination therapy is remarkable, due to the biological characteristics of SCLC, patients are highly susceptible to drug resistance leading to tumor recurrence, with a median overall survival (mOS) of 9–11 months (6, 8, 9). In recent years, the role of immunotherapy in the treatment of ES-SCLC has become increasingly prominent. Several studies have shown that immunotherapy represented by programmed death-1 (PD-1) inhibitor and programmed death- ligand 1 (PD-L1) inhibitors significantly prolonged the mOS and median progression-free survival (mPFS) of ES-SCLC patients (10–13). Toripalimab is a PD-1 inhibitor developed in China and approved for marketing in China in December 2018 and in the US in October 2023. The EXTENTORCH study compared the efficacy and safety of Toripalimab combined with chemotherapy (Etoposide + Carboplatin/Cisplatin, EC) versus chemotherapy in the treatment of ES-SCLC (14). The results showed that compared with chemotherapy, Toripalimab plus chemotherapy prolonged the mOS (14.6 vs 13.3 months, hazard ratio [HR] = 0.8, 95% confidence interval [CI] 0.65-0.98) and mPFS (5.8 vs 5.6 months, HR = 0.67, 95%CI: 0.54-0.82), and the security is controllable.

Although Toripalimab combination chemotherapy extended the survival time of ES-SCLC patients compared with conventional chemotherapy, there is a lack of economic evidence to support its use. Choosing a safe, effective and relatively inexpensive drug not only reduces the economic burden on patients but also facilitates the rational allocation of healthcare resources. Therefore, this study is aims to evaluate the cost-effectiveness of Toripalimab in combination with chemotherapy in the field of first-line treatment of ES-SCLC based on the perspective of the health system in China and the US.

## 2 Methods

### 2.1 Study design

The study was designed and conducted in accordance with the Consolidated Health Economic Evaluation Report Standards (CHEERS) checklist (**Supplementary Table S1**) (15). Study data were obtained from the EXTENTORCH study (ClinicalTrials.gov Identifier: NCT04012606), the Menet (https://www.menet.com.cn/), the Drug. price guide (https://www.drugs.com/price-guide/) and published literature.

The target patients included in this study were consistent with the EXTENTORCH study, that is, age ≥ 18 years and diagnosis of ES-SCLC confirmed by pathology or histology, and more detailed clinical characteristics of the patients are shown in **Supplementary Table S2** (14). The target patients received drug therapy in two groups (1). Toripalimab group:first received 4 cycles (1 cycle = 3 weeks = 21 days) of Toripalimab (240mg, day1) + Etoposide (100mg/m^2^, day1-day3) + Carboplatin (AUC=5mg/mL/min, day1)/Cisplatin (75mg/m^2^, day1), followed by Toripalimab (240mg, day1) until disease progression (2); EC group: first received 4 cycles of Placebo (day1) + Etoposide (100mg/m^2^, day1-day3) + Carboplatin (AUC=5mg/mL/min, day1)/Cisplatin (75mg/m^2^, day1), followed by Placebo (day1) until disease progression. The treatment plan after disease progression provided by the EXTENTORCH study was not detailed, so Topotecan (1.25 mg/m^2^/day, day1-day5) was chosen as the second-line treatment according to the NCCN guideline and the CSCO guideline (16, 17). And best supportive care (BSC) was given to patients not receiving Topotecan. All drugs were given by intravenous route.

### 2.2 Model overview

**Figure 1:**
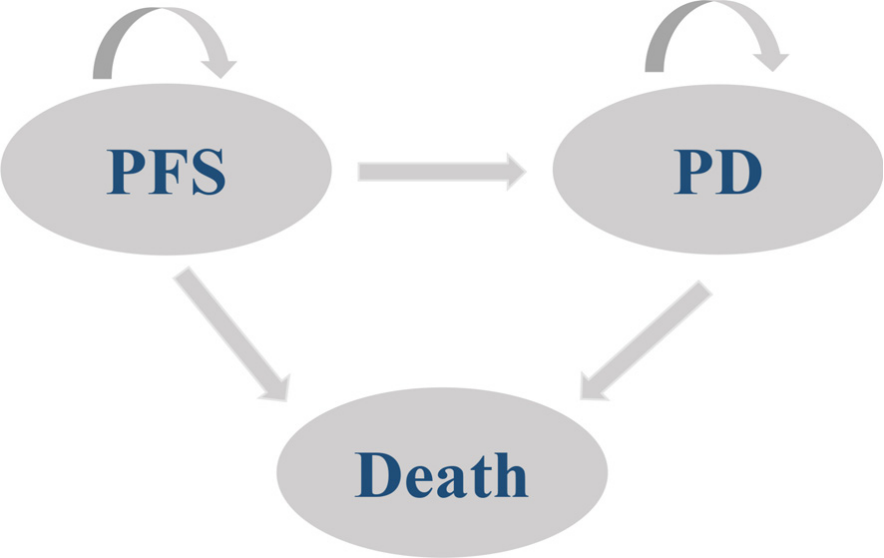
Schematic of the model structure. PFS, Progression-free survival; PD, Progressive disease.

A partitioned survival model was developed to simulate the progression of ES-SCLC by TreeAge Pro software (Version:2022). The model starts from the progression-free survival (PFS) state, and patients in the PFS state can transfer to the PFS state, progressive disease (PD) or the death state, and patients in the PD state can also transfer to the PD state or the death state. The model structure is shown in **Figure 1**. Considering the survival of ES-SCLC patients and the treatment cycle of the EXTENTORCH study, a time horizon (TH) of 10 years with a 21-day modeling cycle was set in this study. The model output metrics included incremental cost-effectiveness ratio (ICER), quality-adjusted life-years (QALYs), incremental QALYs, total cost, and incremental cost.

### 2.3 Survival estimate

The EXTENTORCH study provided survival-related data (14). Firstly, survival information from PFS curves and OS curves in the EXTENTORCH study was digitally extracted using Engauge Digitizer software. The individual data were then reconstructed according to the method by Guyot P et al. (18). The survHE package in R software (4.3.1) (https://www.r-project.org/) was then invoked to fit the parameter distributions of the survival curves. The optimal fitting model was selected by combining the visual inspection with Akaike information criterion (AIC) and Bayesian information criterion (BIC). Smaller values of AIC and BIC indicate better fitting. **Supplementary Table S3** shows the AIC and BIC values of each model, and it can be seen that Log-logistic distribution is the optimal fitting model for all curves. The relevant fitting parameters are shown in **Supplementary Table S4**, and the fitting curve graphs are shown in **Supplementary Figure S1**.

### 2.4 Cost and utility estimate

The perspective was the health system, so only direct healthcare costs were considered, including drug costs, BSC costs, adverse reactions (ADRs) handling costs, hospitalization costs and follow-up costs. Drug cost information was obtained from the Menet and Drug. price guide, and all other costs were obtained from the relevant literature. ADRs treatment costs were only considered for grade 3–4 ADRs with an incidence rate greater than 5%, and it was assumed that ADRs treatment costs were one-time costs. To calculate the drug cycle cost, it was assumed that the body weight of the Chinese patient was 65Kg, the body surface area (BSA) was 1.72m^2^, and the creatinine clearance (CCR) was 70ml/min (19, 20); American patients had a body weight of 70Kg, a BSA of 1.82m^2^, and a CCR of 70ml/min (21, 22). **Tables 1**, **2** provide detailed cost information.

**Table 1:** Model parameters in China.

Variable	Baseline value	Range	Distribution	Source
Cost (US$)				
Toripalimab per 240mg	261.2	208.96~313.44	Gamma	*
Durvalumab per 500mg	2,488.03	1,990.43~2,985.64	Gamma	*
Atezolizumab per 1,200mg	4,511.69	3,609.35~5,414.03	Gamma	*
Etoposide per 100mg	19.55	1.07~61.9	Gamma	*
Carboplatin per 100mg	7.59	7.1~12.63	Gamma	*
Cisplatin per 30mg	2.78	2.63~3.85	Gamma	*
Topotecan per 2mg	14.12	11.29~16.94	Gamma	*
BSC per cycle	345.6	276.48~414.72	Gamma	(26)
Follow-up per cycle	164.73	131.79~197.68	Gamma	(39)
Hospitalization per cycle	61.57	49.25~73.88	Gamma	(39)
Decreased platelet count	1,505.92	1,204.74~1,807.1	Gamma	(40)
Decreased WBC count	115.01	92.01~138.01	Gamma	(40)
Anemia	138.75	111~166.5	Gamma	(40)
Decreased neutrophil count	115.01	92.01~138.01	Gamma	(40)
Hyponatremia	0.29	0.232~0.348	Gamma	(41)
Febrile neutropenia	115.01	92.01~138.01	Gamma	(40)
Incidence of ADRs in Toripalimab group				
Decreased platelet count	24.8%	19.84%~29.76%	Beta	(14)
Decreased WBC count	38.7%	30.96%~46.44%	Beta	(14)
Anemia	30.6%	24.48%~36.72%	Beta	(14)
Decreased neutrophil count	74.3%	59.44%~89.16%	Beta	(14)
Hyponatremia	6.3%	5.04%~7.56%	Beta	(14)
Incidence of ADRs in EC group				
Decreased platelet count	34.3%	27.44%~41.16%	Beta	(14)
Decreased WBC count	44.9%	35.92%~53.88%	Beta	(14)
Anemia	34.7%	27.76%~41.64%	Beta	(14)
Decreased neutrophil count	75%	60%~90%	Beta	(14)
Hyponatremia	6.5%	5.2%~7.8%	Beta	(14)
Incidence of ADRs in DEC group				
Decreased platelet count	6%	4.8%~7.2%	Beta	(13)
Febrile neutropenia	6%	4.8%~7.2%	Beta	(13)
Anemia	9%	7.2%~10.8%	Beta	(13)
Decreased neutrophil count	24%	19.2%~28.8%	Beta	(13)
Incidence of ADRs in AEC group				
Decreased platelet count	10%	8%~12%	Beta	(29)
Decreased WBC count	5%	4%~6%	Beta	(29)
Anemia	14%	11.2%~16.8%	Beta	(29)
Decreased neutrophil count	23%	18.4%~27.6%	Beta	(29)
Utility value				
PFS	0.804	0.536~0.84	Beta	(23)
PD	0.321	0.05~0.473	Beta	(23)
Disutility value				
Decreased platelet count	0.05	0.04~0.06	Beta	(40)
Decreased WBC count	0.2	0.16~0.24	Beta	(23)
Anemia	0.073	0.059~0.089	Beta	(23)
Decreased neutrophil count	0.2	0.16~0.24	Beta	(23)
Hyponatremia	0.09	0.072~0.108	Beta	(41)
Febrile neutropenia	0.2	0.16~0.24	Beta	(23)
Proportion of patients receiving second-line treatment				
Toripalimab group	55.2%	44.16%~66.24%	Beta	(14)
EC group	69.4%	55.52%~83.28%	Beta	(14)
Others				
Discount rate	5%	0~8%	Beta	(24)
Weight	65	52~78	Normal	(19, 20)
Body surface area	1.72	1.376~2.064	Normal	(19, 20)
Creatinine clearance	70	56~84	Normal	(19, 20)

* refers to The Menet (https://www.menet.com.cn/).

BSC, Best supportive care; WBC, White blood cell; ADRs, Adverse reactions; PFS, Progression-free survival; PD, Progressive disease.

**Table 2:** Model parameters in the US.

Variable	Baseline value	Range	Distribution	Source
Cost (US$)				
Toripalimab per 240mg	9,711.6	7,769.28~11,653.92	Gamma	*
Durvalumab per 500mg	4,404.96	3,523.97~5,285.95	Gamma	*
Atezolizumab per 1,200mg	11,756.14	9,404.91~14,107.37	Gamma	*
Etoposide per 100mg	13.68	10.94~16.42	Gamma	*
Carboplatin per 50mg	13.79	11.03~16.55	Gamma	*
Cisplatin per 50mg	15.8	12.64~18.96	Gamma	*
Topotecan per 4mg	80.17	64.14~96.2	Gamma	*
BSC per cycle	1,447.79	1,158.23~1,737.35	Gamma	(26)
Follow-up per cycle	241	192.8~289.2	Gamma	(26)
Hospitalization per cycle	61.57	49.25~73.88	Gamma	(39)
Decreased platelet count	13,105	10,484~15,726	Gamma	(26)
Decreased WBC count	13,105	10,484~15,726	Gamma	(26)
Anemia	7,941	6,352.8~9,529.2	Gamma	(26)
Decreased neutrophil count	13,656	10,924.8~16,387.2	Gamma	(26)
Hyponatremia	0.29	0.232~0.348	Gamma	(41)
Febrile neutropenia	13,656	10,924.8~16,387.2	Gamma	(26)
Incidence of ADRs in Toripalimab group				
Decreased platelet count	24.8%	19.84%~29.76%	Beta	(14)
Decreased WBC count	38.7%	30.96%~46.44%	Beta	(14)
Anemia	30.6%	24.48%~36.72%	Beta	(14)
Decreased neutrophil count	74.3%	59.44%~89.16%	Beta	(14)
Hyponatremia	6.3%	5.04%~7.56%	Beta	(14)
Incidence of ADRs in EC group				
Decreased platelet count	34.3%	27.44%~41.16%	Beta	(14)
Decreased WBC count	44.9%	35.92%~53.88%	Beta	(14)
Anemia	34.7%	27.76%~41.64%	Beta	(14)
Decreased neutrophil count	75%	60%~90%	Beta	(14)
Hyponatremia	6.5%	5.2%~7.8%	Beta	(14)
Incidence of ADRs in DEC group				
Decreased platelet count	6%	4.8%~7.2%	Beta	(13)
Febrile neutropenia	6%	4.8%~7.2%	Beta	(13)
Anemia	9%	7.2%~10.8%	Beta	(13)
Decreased neutrophil count	24%	19.2%~28.8%	Beta	(13)
Incidence of ADRs in AEC group				
Decreased platelet count	10%	8%~12%	Beta	(29)
Decreased WBC count	5%	4%~6%	Beta	(29)
Anemia	14%	11.2%~16.8%	Beta	(29)
Decreased neutrophil count	23%	18.4%~27.6%	Beta	(29)
Utility value				
PFS	0.84	0.536~0.84	Beta	(23)
PD	0.166	0.05~0.473	Beta	(23)
Disutility value				
Decreased platelet count	0.05	0.04~0.06	Beta	(40)
Decreased WBC count	0.2	0.16~0.24	Beta	(23)
Anemia	0.073	0.059~0.089	Beta	(23)
Decreased neutrophil count	0.2	0.16~0.24	Beta	(23)
Hyponatremia	0.09	0.072~0.108	Beta	(41)
Febrile neutropenia	0.2	0.16~0.24	Beta	(23)
Proportion of patients receiving second-line treatment				
Toripalimab group	55.2%	44.16%~66.24%	Beta	(14)
EC group	69.4%	55.52%~83.28%	Beta	(14)
Others				
Discount rate	3%	0~8%	Beta	(25)
Weight	65	52~78	Normal	(21, 22)
Body surface area	1.72	1.376~2.064	Normal	(21, 22)
Creatinine clearance	70	56~84	Normal	(21, 22)

* refers to The Drug. price guide (https://www.drugs.com/price-guide/).

BSC, Best supportive care; WBC, White blood cell; ADRs, Adverse reactions; PFS, Progression-free survival; PD, Progressive disease.

The utility value information was extracted from an international study, which showed that Chinese patients had a utility value of 0.804 in the PFS stage and 0.321 in the PD stage, whereas American patients had a utility value of 0.84 in the PFS stage and 0.166 in the PD stage (23). The reduction in the utility value due to ADRs was also obtained from the published literature, and the specific information on the utility value is shown in **Tables 1**, **2**.

When the TH is more than 1 year, the costs and health outputs occurring in the future need to be discounted. The TH of this study is 10 years, so an annual discount rate of 5% was taken for China and 3% for the United States based on relevant guidelines and literature recommendations (24, 25).

### 2.5 Basic analysis

The economy of Toripalimab regimen was judged by comparing the ICER with the willingness to pay (WTP), if the ICER is greater than the WTP, it is considered that Toripalimab regimen does not have cost-effective advantage, and conversely, it is cost-effective. According to the literature and guideline recommendations, the WTP for China was set to be 3 times the gross domestic product (GDP) per capita in 2023 (WTP = 3*GDP = 3*$12,291 = $36,874/QALY) for the basic analysis, whereas that for the US was set to be $150,000/QALY (24, 26)

### 2.6 Sensitivity analysis

One-way sensitivity analysis (OWSA) was performed to investigate the effect of single parameter changes on the model, and the results was presented as a tornado plot. The Menet provides a range of values for the cost parameter, published literature provides a range of values for the utility value, the discount rate is recommended to be set at 0-8% according to the guideline, and the ranges of the other parameters are set at± 20% of their base values (24, 26).

The effects of simultaneous changes in multiple parameters on the model were examined by probabilistic sensitivity analysis (PSA), and the results were presented as a cost-effectiveness scatterplot and a cost-effectiveness acceptability curve (CEAC). Second-order Monte Carlo simulations were used to perform PSA for 1,000 random repeated samples of parameters conforming to different probability distributions. In this study, the costs obeyed the Gamma distribution, and the utility values, the incidence of ADRs, and the discount rate obeyed the Beta distribution.

### 2.7 Scenario analysis

Scenario Analysis 1:In order to explore the effect of different TH on the results, the TH was set to 5 and 8 years for the scenario analysis, respectively.

Scenario Analysis 2:Health utility values are often one of the most important causes of variation in the results of pharmacoeconomic evaluations (27). To assess the impact of utility values on outcomes, Scenario Analysis 2 used the results of a real-world study for a cost-effectiveness analysis with utility values of 0.7 for the PFS stage and 0.6 for the PD stage (28).

### 2.8 Exploratory analysis

The two internationally recognized first-line immunotherapy regimens for ES-SCLC are Durvalumab plus EC (DEC) and Atezolizumab plus EC (AEC). This exploratory analysis aims to compare the cost-effectiveness of DEC/AEC vs Toripalimab combined with EC (TEC) in the treatment of ES-SCLC. The CASPIAN and IMpower133 trials provided therapeutic and survival information for ES-SCLC patients (13, 29). Firstly, given the absence of head-to-head clinical trials of DEC/AEC vs TEC, this study designated TEC as the control group and employed network meta-analysis (NMA) to construct a comparative framework. This approach enabled the estimation of hazard ratios (HRs) of PFS and OS for DEC/AEC vs TEC, and the detailed results are presented in **Supplementary Table S5**. Subsequently, referencing the methodology proposed by Hoyle et al. in the pharmacoeconomic evaluation of advanced renal cancer, the following parametric transformation formula was applied to adjust and calibrate the survival data of DEC group and AEC group (30).


$$\gamma_{comparator\;drug}=\;\gamma_{control\;drug},\;\lambda_{comparison\;drug}=\;\lambda_{control\;drug}*\;HR$$

Finally, the costs of DEC group and AEC group were calculated in accordance with the methodology outlined in Section 2.4 (“Cost and Utility Estimate”). By integrating costs and survival information, models can be established to perform a comprehensive cost-effectiveness of TEC vs DEC/AEC in the treatment of ES-SCLC.

## 3 Results

### 3.1 Results of basic analysis

The results of the basic analysis showed that not only were the total costs higher in the Toripalimab group than in the EC group, both in China and in the US ($16,714 vs $11,510, $57,561 vs $ 214,484), but the health outputs were also more than in the EC group (0.93 QALYs vs 0.75 QALYs, 0.79 QALYs vs 0.62 QALYs). Toripalimab regimen was cost-effective in China (ICER: $29,139/QALY< WTP: $36,874/QALY) but not in the US (ICER: $915,965/QALY > WTP: $150,000/QALY). See **Table 3** for details of the results.

**Table 3:** Results of basic analysis.

Variable	Cost ($)	Incremental cost ($)	QALYs	Incremental QALYs	ICER($/QALY)
China					
EC group	11,510		0.75		
Toripalimab group	16,714	5,204	0.93	0.18	29,139
US					
EC group	57,561		0.62		
Toripalimab group	214,484	156,923	0.79	0.17	915,965

QALYs, Quality-adjusted life years; ICER, Incremental cost-effectiveness ratio.

### 3.2 Results of sensitivity analysis

**Figure 2:**
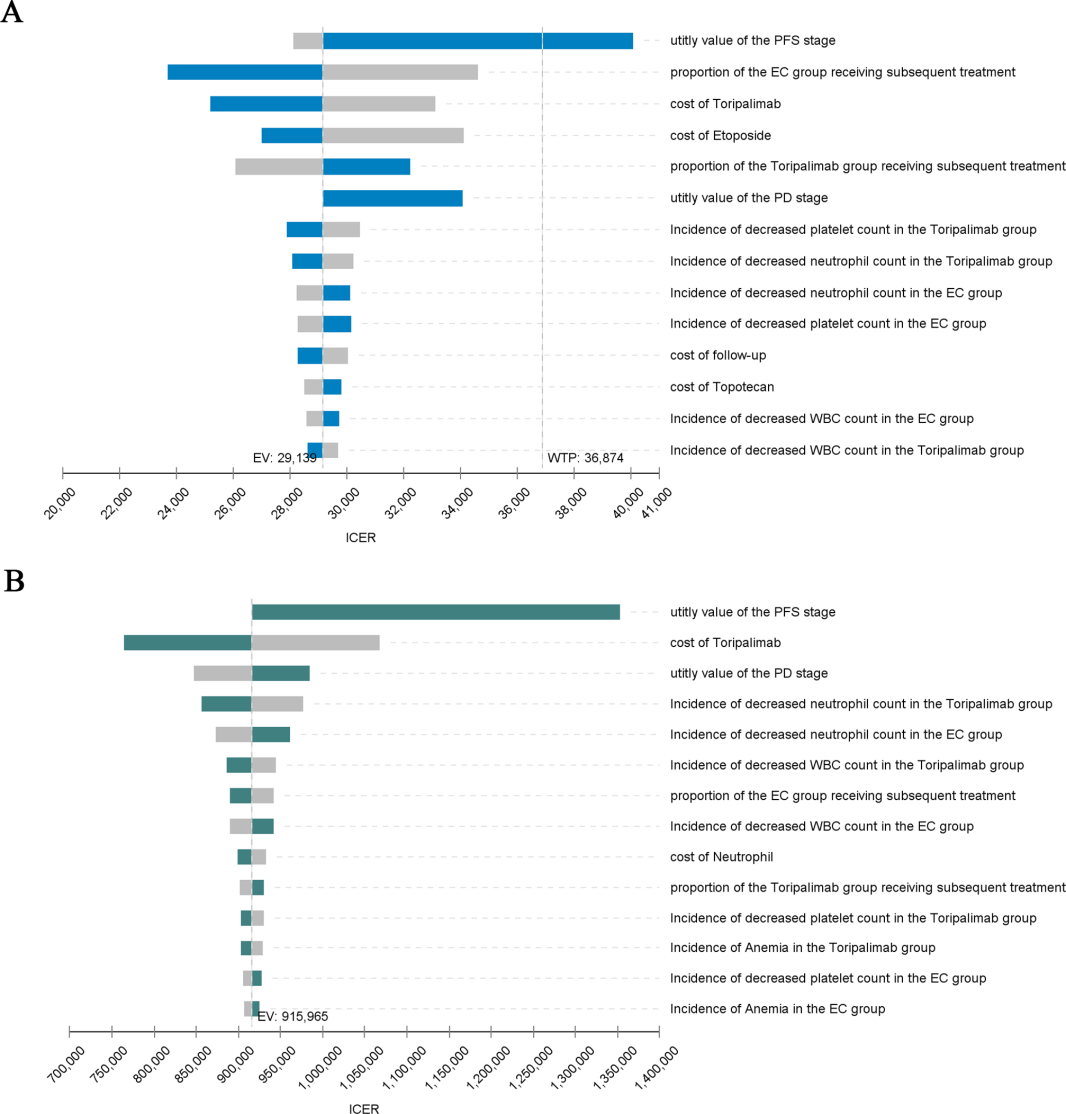
Tornado Diagram. **(A)** China: Tornado Diagram. **(B)** America: Tornado Diagram. PFS, Progression-free survival; PD, Progressive disease; ICER, Incremental cost-effectiveness ratio; EV, Expected value; WTP, Willingness to pay; WBC, White blood cell.

The results of OWSA are shown in **Figure 2**. As can be seen from the tornado plot, in China, the utility value of the PFS stage had the greatest impact on ICER, and the proportion of patients in the EC group receiving second-line therapy, the cost of Toripalimab and Etoposide, and the utility value of the PD stage also had a moderate impact on ICER. In the US, the parameters that had the greatest impact on ICER included the utility value of the PFS stage and the cost of Toripalimab, and the other parameters had a small impact on ICER.

**Figure 3:**
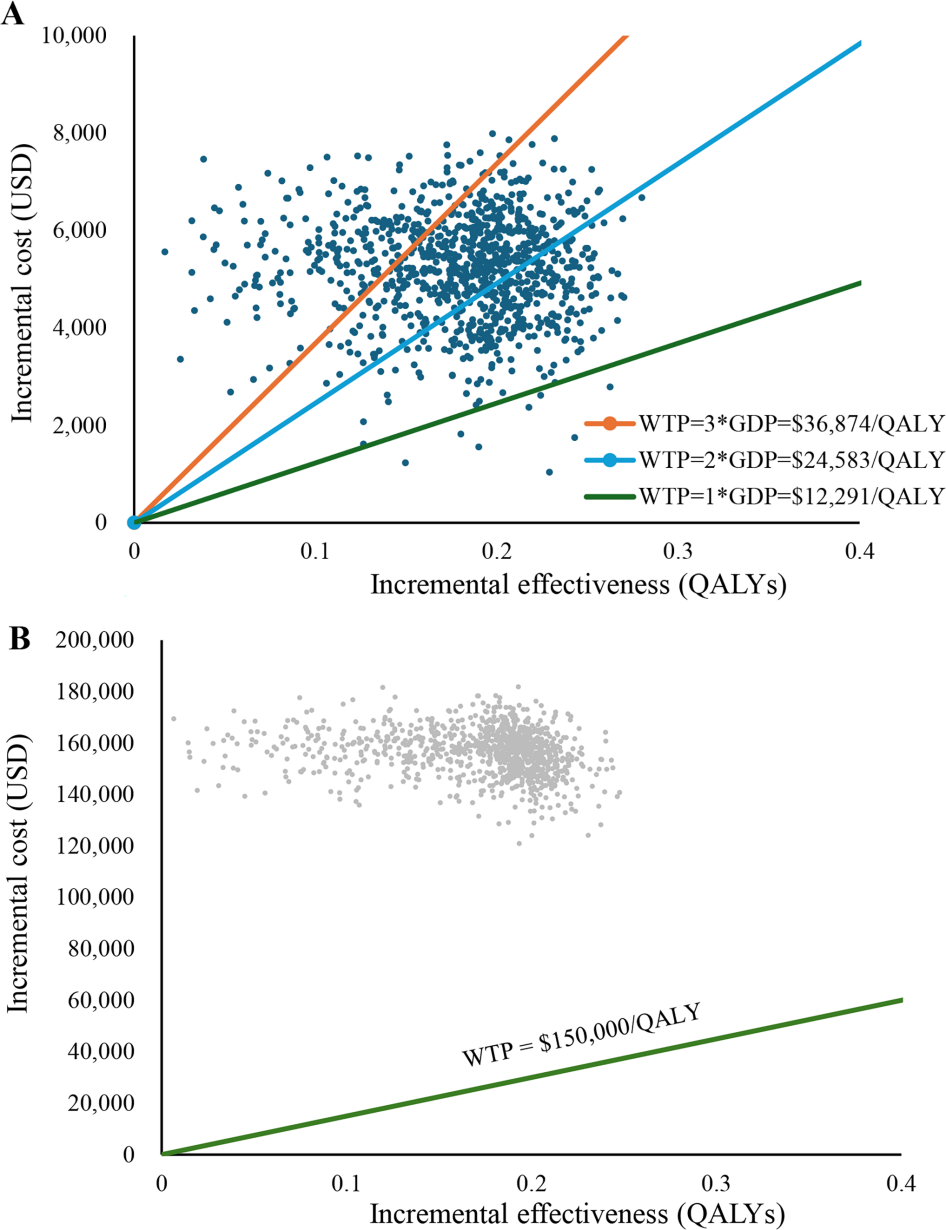
Cost-effectiveness scatter plot. **(A)** China: Cost-effectiveness scatter plot. **(B)** America: Cost-effectiveness scatter plot. WTP, Willingness to pay; GDP, gross domestic product; QALY, Quality-adjusted life year.

The results of PSA are shown in **Figures 2**, **3**, respectively. The cost-effectiveness scatter plot for China shows that when WTP is set to 1*GDP, 2*GDP and 3*GDP, the economic probabilities of Toripalimab scheme are 0.7%, 29.7% and 77.1%, respectively. However, the cost-effectiveness scatter plot for America shows that Toripalimab is unlikely to be economical even if WTP is set at the current high value ($150,000/QALY). The CEAC chart demonstrates that the Toripalimab regimen begins to demonstrate potential economic advantage (probability = 0.1%) at a WTP threshold of $6,667/QALY in the Chinese healthcare context. Conversely, in the U.S. healthcare system, a significantly higher WTP threshold of $540,000/QALY is required for the Toripalimab regimen to demonstrate comparable economic viability. In China, the Toripalimab regimen attains a 50.5% probability of cost-effectiveness at a WTP threshold of $28,400/QALY, thereby surpassing conventional chemotherapy in economic efficiency. By contrast, within the US healthcare paradigm, the Toripalimab regimen necessitates an exceptionally high WTP threshold ($867,000/QALY) to achieve equivalent cost-effectiveness probability (50%) relative to conventional chemotherapy approaches.

### 3.3 Results of scenario analysis

**Table 4** provides the results of the detailed scenario analysis. Scenario analysis 1 shows that the Toripalimab regimen is economical in China, but not in the US, regardless of whether the TH is 5 or 8 years. Scenario analysis 2 shows that after changing the utility value, the ICER decreases to $28,199/QALY (<WTP) in China and to $850,350/QALY (> WTP) in the US.

**Table 4:** Results of scenario analysis and exploratory analysis.

Variable	Cost ($)	Incremental cost ($)	QALYs	Incremental QALYs	ICER($/QALY)
Scenario Analysis 1: TH = 5 years					
China					
EC group	11,186		0.73		
Toripalimab group	16,018	4,831	0.89	0.16	30,341
US					
EC group	56,802		0.61		
Toripalimab group	211,082	154,281	0.77	0.16	983,320
Scenario Analysis 1: TH = 8 years					
China					
EC group	11,439		0.75		
Toripalimab group	16,552	5,113	0.92	0.17	29,403
US					
EC group	57,395		0.62		
Toripalimab group	213,703	156,308	0.79	0.17	931,041
Scenario Analysis 2					
China					
EC group	11,510		0.96		
Toripalimab group	16,714	5,204	1.14	0.18	28,199
US					
EC group	57,561		0.96		
Toripalimab group	214,484	156,923	1.14	0.18	850,350
Exploratory Analysis					
China:DEC VS TEC					
TEC group	16,714		0.93		
DEC group	111,159	94,445	1.00	0.07	1,368,881
China:AEC VS TEC					
TEC group	16,714		0.93		
AEC group	82,674	65,960	0.96	0.04	1,808,957
US:DEC VS TEC					
TEC group	214,484		0.79		
DEC group	215,910	1,426	0.90	0.11	12,928
US:AEC VS TEC					
TEC group	214,484		0.79		
AEC group	226,134	11,650	0.88	0.09	125,496

QALYs, Quality-adjusted life years; ICER, Incremental cost-effectiveness ratio.

### 3.4 Results of exploratory analysis

The exploratory analysis (**Table 2**) indicates that TEC demonstrates cost-effectiveness for ES-SCLC treatment compared with DEC/AEC within the Chinese healthcare system (ICER_DEC vs TEC_: $1,368,881/QALY, ICER_AEC vs TEC_: $1,808,957/QALY), whereas it fails to demonstrate economic viability in the US context (ICER_DEC vs TEC_: $$12,928/QALY, ICER_AEC vs TEC_: $125,496/QALY).

## 4 Discussion

Recent years have witnessed significant advancements in the clinical application of immunotherapy for ES-SCLC. Currently, DEC and AEC regimens have been established as internationally recognized first-line immunotherapy standards for ES-SCLC. On the one hand, comparative pricing analysis reveals that Toripalimab demonstrates cost advantages over both Durvalumab and Atezolizumab in pharmaceutical markets across China and the US. T The cost of Toripalimab ($261.20/cycle) was significantly lower than that of Atezolizumab ($4,511.69/cycle) and Durvalumab ($7,464.10/cycle) in the Chinese market. Similarly, Toripalimab ($9,711.60/cycle) also offers a price advantage over Atezolizumab ($11,756.14/cycle) and Durvalumab ($13,214.88/cycle) in the U.S. market. On the other hand, the EXTENTORCH trial demonstrated survival benefits with Toripalimab regimen compared to chemotherapy alone. This combination of clinical efficacy and price advantage strongly suggests that Toripalimab regimen may have a unique cost-effectiveness advantage. Existing studies have consistently demonstrated the AEC and DEC regimens are not cost-effective in the treatment of ES-SCLC in China and the US (31–34). However, no study has evaluated the cost-effectiveness of TEC regimen in the treatment of ES-SCLC. Considering the price advantage and clinical benefits of Toripalimab, a systematic pharmacoeconomic assessment of Toripalimab regimen carries important clinical and policy implications. Therefore, this study evaluated the cost-effectiveness of TEC regimen for ES-SCLC from the perspective of health systems in China and the US. The results showed that Toripalimab regimen was economical in China and uneconomical in the US when compared with either chemotherapy or AEC/DEC regimen.

OWSA showed that the health utility value at the PFS stage was the parameter that had the greatest impact on ICER in both the Chinese and American perspectives. The utility value directly affects the calculation of QALY, which is a key indicator for assessing the effectiveness of medical interventions. The utility value expresses the quality of life between full health and death, and the QALY allows the conversion of life years of different interventions into equivalent years of health status, thus facilitating the comparison of costs and effects between different treatment options. In the cost-effectiveness analysis, too high utility value leads to a relatively low ICER. Conversely, if the utility value is underestimated, the ICER will be higher. Therefore, choosing an appropriate utility value is crucial for calculating ICER. Unfortunately, the EXTENTORCH study did not report the utility value data, and there is a lack of research on the quality of life (i.e., utility value) of patients with SCLC. For the basic analysis, this study referred to published studies and chose to use the results of a large international study on the utility value in non-small cell lung cancer as an alternative to the utility value for SCLC (31, 32). But in scenario analysis 2, this study also changed the health utility values for PFS stage and PD stage based on the results of a study on the quality of life of SCLC patients (28). Considering the small sample size included in that real-world study, the results may not be representative enough to be analyzed as a special scenario only. Fortunately, scenario analysis 2 was consistent with the findings of the basic analysis.

PSA not only elucidated the economic disparities of the Toripalimab regimen across different geographical contexts, but also provided a quantifiable comparison basis for cross-regional investigations through specific WTP thresholds and corresponding economic probabilities. The China Guidelines for Pharmacoeconomic Evaluation (2020) recommend using 1~3 times the national GDP per capita as the WTP (24). When performing PSA, this study sets WTP to 1*GDP, 2*GDP, and 3*GDP respectively. The analytical outcomes revealed that across 1,000 simulations, he Toripalimab regimen demonstrated cost-effectiveness probabilities of 0.7%, 29.7%, and 77.1%, respectively, under these progressive WTP thresholds, thereby indicating substantial sensitivity of the regimen’s economic viability to WTP parameters within the Chinese healthcare context. From the perspective of the US healthcare system, where cost-effectiveness evaluations for oncological interventions typically employ WTP thresholds ranging from $100,000 to $150,000/QALY (35). In this study, the Toripalimab regimen failed to demonstrate economic viability even at a high WTP of $150,000/QALY gained. This observation potentially reflects an incongruity between the pricing structure of Toripalimab and its corresponding clinical benefit profile within the US healthcare framework.

Given that TH also has a significant impact on ICER, scenario analysis 1 calculated ICER under different TH (36). Typically, the TH for pharmacoeconomic evaluation should be sufficiently long to obtain the full impact of the intervention measures on the costs and health outcomes of the subjects (24, 37). When fitting and extrapolating the survival curves, we found that the mortality rate in the Toripalimab group reached 99% when the TH was set to 10 years, so a TH of 10 years was chosen for the basic analysis. We also found that the mortality rate in the EC group had reached 99% when the TH = 8 years, so the ICER at TH = 8 years was also calculated in scenario analysis 1. Considering that the ES-SCLC is extremely malignant and the 5-year survival rate of patients receiving EC therapy is also low, so a TH of 5 years was also set. Previously Kim et al. found that the ICER decreases with increasing TH in most of the pharmacoeconomically evaluated studies (36). Scenario analysis 1 also showed that incremental costs and incremental QALYs increase with increasing TH, but the advantage of increasing incremental QALYs is sufficient to compensate for the disadvantage of increasing incremental costs, so it leads to smaller ICER, which confirms the findings of Kim et al. The costs of immunotherapy are mostly incurred in the short term, but because of the “delayed effect” of immunosuppressants, it takes a relatively long time for their health benefits to be realized (36, 38). Therefore, the longer the TH, the more QALYs are captured, leading to a reduction in ICER.

There are also some limitations of this study (1). There are some inevitable biases when survival curves fitting and extrapolation methods are used to obtain survival information beyond the observation period (2). Due to the lack of studies on health utility values in ES-SCLC patients, the use of alternative utility values may have slightly affected the results (3). Given the limited treatment pathway details reported in the EXTENTORCH trial, this study unified subsequent anticancer therapy, which may be different from clinical practice (4). The data utilized for this exploratory analysis were not derived from “head-to-head” clinical trials. Despite the implementation of methodologically rigorous NMA to adjust and calibrate survival data, the indirect nature of these comparisons introduces inherent methodological uncertainties (5). The enrollment population of the EXTENTORCH study did not include U.S. ES-SCLC patients, which may be somewhat different from the actual survival rate of U.S. ES-SCLC. Despite these limitations, this study also verified the stability of the model through sensitivity and scenario analyses, so the results of the study can still provide economic references for policy makers, clinicians and patients.

## 5 Conclusions

This study conducted a comprehensive cost-effectiveness analysis comparing Toripalimab plus chemotherapy versus chemotherapy alone as first-line treatment for ES-SCLC from the dual perspectives of Chinese and US healthcare systems through a modeling approach using data from a phase III clinical trial, and validated the stability of the model through a sensitivity analysis with a series of scenario analyses. The findings demonstrated Toripalimab combination chemotherapy was economic in China when compared to chemotherapy alone, while was uneconomic in the US.

## Data Availability

The original contributions presented in the study are included in the article/**Supplementary Material**. Further inquiries can be directed to the corresponding authors.
