# Supplementary material for: Cost-effectiveness analysis of Toripalimab regimen for extensive-stage small-cell lung cancer in China and America
Source: Front Immunol. 2025 May 16;16:1556100. doi: 10.3389/fimmu.2025.1556100 (PMC12122439; doi:10.3389/fimmu.2025.1556100)
Supplement: Supplementary file 1 [file DataSheet1.docx]

Supplementary Material

Cost-effectiveness analysis of Toripalimab regimen for extensive-stage small cell lung cancer in China and America

**Jing Yang^1^**^†^**, Fang Chen^2^**^†^**,** **Junlin Li^3^**^†^**, Yujie Zhou^4^, Hao Wang^5*,^ Yunchun Long^6*^**

**Authors:**

Jing Yang: mirroryang2020@163.com

Fang Chen: fangchen@xmu.edu.cn

Junlin Li: 13550150790@163.com
Yujie Zhou: yujiezhoum@163.com
^†^These authors have contributed equally to this work and share the first authorship.

**Corresponding Authors:**

Hao Wang: halsey_glyy@163.com

Yunchun Long: chrrich9854@163.com

**Correspondence to:**

Hao Wang

No.321 Zhongshan Road, Nanjing, Jiangsu Province, China

18551668218

halsey_glyy@163.com

Yunchun Long

No. 639 Longmian Avenue, Nanjing, Jiangsu Province, China

17882138834

chrrich9854@163.com

**Affiliations:**

^1^ Department of Pharmacy, Chengdu Second People's Hospital, Chengdu, Sichuan Province, China

^2^ Department of Pharmacy, The First Affiliated Hospital of Xiamen University, Xiamen, Fujian Province, China

^3^ Department of Pharmacy, Nanan People's Hospital of Chongqing, Chongqing, China

^4^ Department of Respiratory and Critical Care Medicine, Nanjing Drum Tower Hospital, Nanjing, Jiangsu Province, China

^5^ Department of Pharmacy, Nanjing Drum Tower Hospital, Nanjing, Jiangsu Province, China

^6^ School of Basic Medicine and Clinical Pharmacy, China Pharmaceutical University, Nanjing, Jiangsu Province, China

# Supplementary Figures and Tables

## Supplementary Figures

**Figure S1.** Kaplan-Meier Curves Fitting and Extrapolation.

PFS: Progression-free disease, OS: Overall survival, K-M: Kaplan-Meier


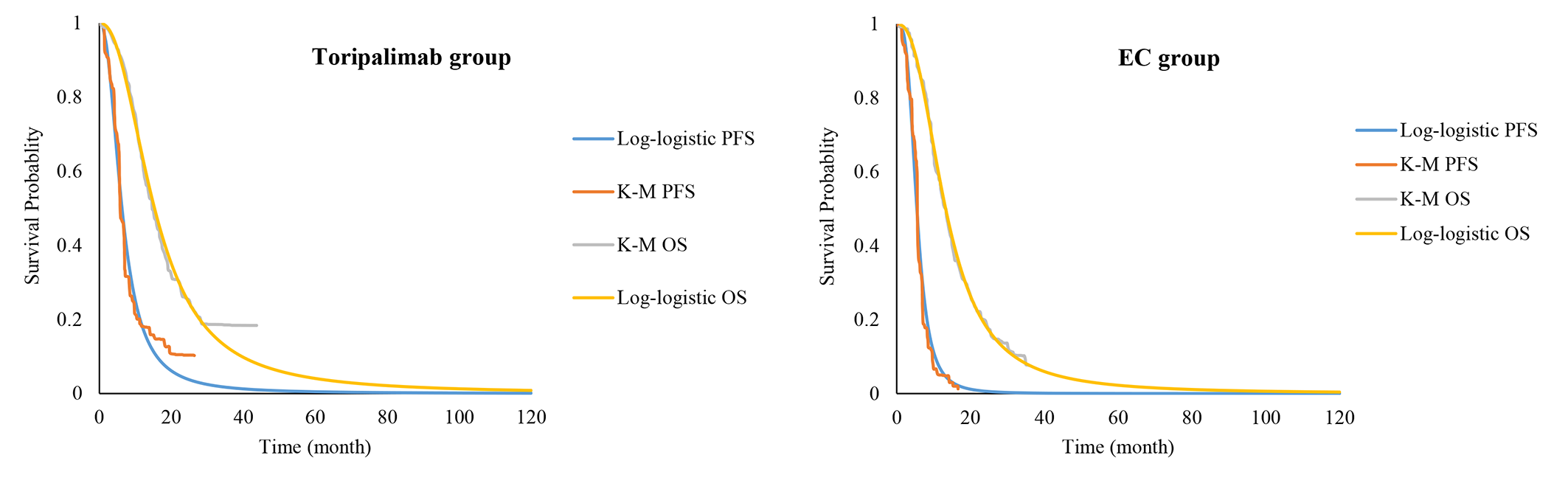


**Figure S2.** Cost-effectiveness acceptability curve. (A). China: Cost-effectiveness acceptability curve. (B). Cost-effectiveness acceptability curve. QALY: Quality-adjusted life year


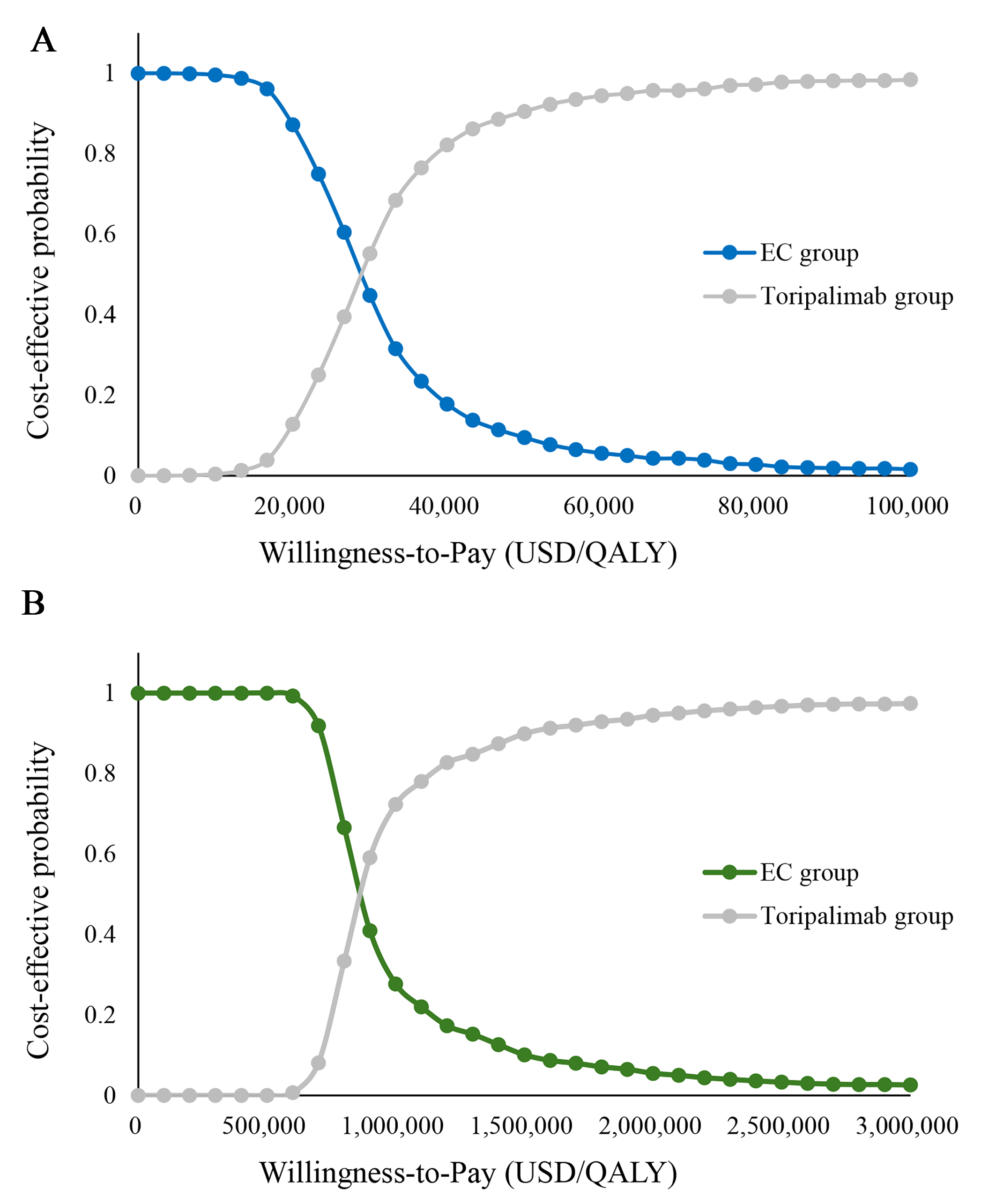


## Supplementary Tables

**Table S1.** CHEERS checklist—Items to include when reporting economic evaluations of health interventions

| **Section/item** | **Item No** | **Recommendation** | **Reported on page No** |
| --- | --- | --- | --- |
| **Title and abstract** | | | |
| Title | 1 | Identify the study as an economic evaluation or use more specific terms such as “cost-effectiveness analysis”, and describe the interventions compared. | 1 |
| Abstract | 2 | Provide a structured summary of objectives, perspective, setting, methods (including study design and inputs), results (including base case and uncertainty analyses), and conclusions. | 2 |
| **Introduction** | | | |
| Background and objectives | 3 | Provide an explicit statement of the broader context for the study. | 3 |
|  |  | Present the study question and its relevance for health policy or practice decisions. | 3 |
| **Methods** | | | |
| Target population and subgroups | 4 | Describe characteristics of the base case population and subgroups analyzed, including why they were chosen. | 4 |
| Setting and location | 5 | State relevant aspects of the system(s) in which the decision(s) need(s) to be made. | 4 |
| Study perspective | 6 | Describe the perspective of the study and relate this to the costs being evaluated. | 4 |
| Comparators | 7 | Describe the interventions or strategies being compared and state why they were chosen. | 4 |
| Time horizon | 8 | State the time horizon(s) over which costs and consequences are being evaluated and say why appropriate. | 4 |
| Discount rate | 9 | Report the choice of discount rate(s) used for costs and outcomes and say why appropriate. | 5 |
| Report the choice of discount rate(s) used for costs and outcomes and say why appropriate. | 10 | Describe what outcomes were used as the measure(s) of benefit in the evaluation and their relevance for the type of analysis performed. | 4 |
| Measurement of effectiveness | 11a | Single study-based estimates: Describe fully the design features of the single effectiveness study and why the single study was a sufficient source of clinical effectiveness data. | Not applicable |
|  | 11b | Synthesis-based estimates: Describe fully the methods used for identification of included studies and synthesis of clinical effectiveness data. | 4 |
| Measurement and valuation of preference based outcomes | 12 | If applicable, describe the population and methods used to elicit preferences for outcomes. | Not applicable |
| Estimating resources and costs | 13a | Single study-based economic evaluation: Describe approaches used to estimate resource use associated with the alternative interventions. Describe primary or secondary research methods for valuing each resource item in terms of its unit cost. Describe any adjustments made to approximate to opportunity costs. | Not applicable |
|  | 13b | Model-based economic evaluation: Describe approaches and data sources used to estimate resource use associated with model health states. Describe primary or secondary research methods for valuing each resource item in terms of its unit cost. Describe any adjustments made to approximate to opportunity costs. | 4, 5 |
| Currency, price date, and conversion | 14 | Report the dates of the estimated resource quantities and unit costs. Describe methods for adjusting estimated unit costs to the year of reported costs if necessary. Describe methods for converting costs into a common currency base and the exchange rate | 5 |
| Choice of model | 15 | Describe and give reasons for the specific type of decision-analytical model used. Providing a figure to show model structure is strongly recommended. | 4 |
| Assumptions | 16 | Describe all structural or other assumptions underpinning the decision-analytical model. | 4-6 |
| Analytical methods | 17 | Describe all analytical methods supporting the evaluation. This could include methods for dealing with skewed, missing, or censored data; extrapolation methods; methods for pooling data; approaches to validate or make adjustments (such as half cycle corrections) to a model; and methods for handling population heterogeneity and uncertainty. | 4-6 |
| **Results** | | | |
| Study parameters | 18 | Report the values, ranges, references, and, if used, probability distributions for all parameters. Report reasons or sources for distributions used to represent uncertainty where appropriate. Providing a table to show the input values is strongly recommended. | 12-15 |
| Incremental costs and outcomes | 19 | For each intervention, report mean values for the main categories of estimated costs and outcomes of interest, as well as mean differences between the comparator groups. If applicable, report incremental cost-effectiveness ratios. | 16 |
| Characterizing uncertainty | 20a | Single study-based economic evaluation: Describe the effects of sampling uncertainty for the estimated incremental cost and incremental effectiveness parameters, together with the impact of methodological assumptions (such as discount rate, study perspective). | Not applicable |
|  | 20b | Model-based economic evaluation: Describe the effects on the results of uncertainty for all input parameters, and uncertainty related to the structure of the model and assumptions. | 6 |
| Characterizing heterogeneity | 21 | If applicable, report differences in costs, outcomes, or cost-effectiveness that can be explained by variations between subgroups of patients with different baseline characteristics or other observed variability in effects that are not reducible by more information. | Not applicable |
| **Discussion** | | | |
| Study findings, limitations, generalizability, and current knowledge | 22 | Summaries key study findings and describe how they support the conclusions reached. Discuss limitations and the generalizability of the findings and how the findings fit with current knowledge. | 7-8 |
| **Other** | | | |
| Source of funding | 23 | Describe how the study was funded and the role of the funder in the identification, design, conduct, and reporting of the analysis. Describe other non-monetary sources of support. | 9 |
| Conflicts of interest | 24 | Describe any potential for conflict of interest of study contributors in accordance with journal policy. In the absence of a journal policy, we recommend authors comply with International Committee of Medical Journal Editors recommendations. | 9 |

For consistency, the CHEERS statement checklist format is based on the format of the CONSORT statement checklist.

**Table S2** Baseline characteristics of patients in EXTENTORCH

|  | Toripalimab group (N = 223) | EC group (N = 219) |
| --- | --- | --- |
| Age, years | | |
| Median (range) | 62.0 (27–80) | 63.0 (30–77) |
| <65 years, n (%) | 144 (64.6) | 124 (56.6) |
| ≥65 years, n (%) | 79 (35.4) | 95 (43.4) |
| Sex, n (%) | | |
| Male | 183 (82.1) | 183 (83.6) |
| Female | 40 (17.9) | 36 (16.4) |
| ECOG performance status, n (%) | | |
| 0 | 42 (18.8) | 38 (17.4) |
| 1 | 181 (81.2) | 181 (82.6) |
| Smoking status, n (%) | | |
| Never | 48 (21.5) | 49 (22.4) |
| Current smoker | 30 (13.5) | 35 (16.0) |
| Former | 145 (65.0) | 135 (61.6) |
| Site of metastasis | | |
| Liver | 60 (26.9) | 50 (22.8) |
| Brain | 3 (1.3) | 4 (1.8) |
| Programmed cell death ligand 1 expression | | |
| Tumor cell < 1% | 152 (68.2) | 141 (64.4) |
| Tumor cell≥ 1% | 20 (9.0) | 29 (13.2) |
| Unknown | 51 (22.9) | 49 (22.4) |

*Previous therapy’ refers to the treatment previously for limited-stage SCLC

Note: This table was extracted from the EXTENTORCH trial. Content of this table reflected the baseline characteristics of patients with extensive-stage small cell lung cancer in the EXTENTORCH trial.

**Table S3.** Goodness-of-fit results

|  | Exponential | Weibull | Gompertz | Log-logistic | Log-normal | Gamma |
| --- | --- | --- | --- | --- | --- | --- |
| PFS of Toripalimab group | | | | | | |
| AIC | 1,080.214 | 1,047.004 | 1,076.479 | **1,012.171** | 1,020.426 | 1,033.646 |
| BIC | 1,083.621 | 1,053.818 | 1,083.293 | **1,018.986** | 1,027.24 | 1,040.46 |
| PFS of EC group | | | | | | |
| AIC | 1,078.2353 | 949.762 | 1,000.03 | **935.301** | 953.3887 | 940.1982 |
| BIC | 1,081.6243 | 956.540 | 1,006.808 | **942.079** | 960.1668 | 946.9764 |
| OS of Toripalimab group | | | | | | |
| AIC | 1,388.788 | 1,345.843 | 1,373.652 | **1,324.746** | 1,331.555 | 1,336.418 |
| BIC | 1,392.195 | 1,352.658 | 1,380.466 | **1,331.562** | 1,338.369 | 1,343.233 |
| OS of EC group | | | | | | |
| AIC | 1,420.431 | 1,361.386 | 1,391.329 | **1,343.856** | 1,347.472 | 1,351.286 |
| BIC | 1,423.82 | 1,368.164 | 1,398.107 | **1,350.634** | 1,354.25 | 1,358.064 |

PFS: Progression-free survival, OS: Overall survival, AIC: Akaike Information Criterion, BIC: Bayesian Information Criterion.

**Table S4**. Fitted parameter

| Variable | Optimal distribution function | Baseline value |
| --- | --- | --- |
| PFS curve | | |
| Toripalimab group | Log-logistic | γ: 2.35,398, λ: 6.27,432 |
| EC group | Log-logistic | γ: 3.40,618, λ: 5.45,408 |
| OS curve | | |
| Toripalimab group | Log-logistic | γ: 2.31,447, λ: 15.31,918 |
| EC group | Log-logistic | γ: 2.49,814, λ: 13.26,107 |
| PFS: Progression-free survival, OS: Overall survival, γ: shape parameter, λ: scale parameter | | |

**Table S5**. Results of network meta-analysis

| Group | HR |
| --- | --- |
| DEC vs TEC: PFS | 1.2 |
| DEC vs TEC: OS | 0.94 |
| AEC vs TEC: PFS | 1.2 |
| AEC vs TEC: OS | 0.88 |

FS: Progression-free survival, OS: Overall survival, HR: Hazard ratios
